# Ossified Spinal Meningioma: A Case Report and a Review of the Literature

**DOI:** 10.1055/s-0039-1697634

**Published:** 2019-10-01

**Authors:** Mahmoud M. Taha, Ahmed Alawamry, Hesham R. Abdel-Aziz

**Affiliations:** 1 Department of Neurosurgery, Faculty of Medicine, Zagazig University, Zagazig, Egypt; 2 Department of Pathology, Faculty of Medicine, Zagazig University, Zagazig, Egypt

**Keywords:** ossified meningioma, spinal meningioma, intradural extramedullary

## Abstract

Ossified spinal meningiomas are a rare form of spinal tumors. These tumors increase surgical morbidities due to their hard consistency and strong adhesion to the neural tissue and relatively narrow surgical space. Here, the authors describe the clinical findings, surgical strategies, and histological findings of a patient with an ossified meningioma. Preoperative diagnosis of these tumors can prevent surgical morbidities. Total resection can be curative with the application of meticulous microsurgical techniques.

Metaplastic meningiomas are a subtype of meningioma characterized by focal mesenchymal differentiation with osseous, cartilaginous, lipomatous, myxoid, or xanthomatous changes. 1 These tumors are most commonly located at intradural extramedullary sites (40% of cases). 2 Ossified meningiomas account for 0.7 to 5.5% of all spinal meningiomas. 3 Symptoms are common, and surgical resection is a technically demanding procedure due to the hard consistency of these tumors and spatial restriction during surgery for spinal tumors. 4

## Case Report

**Fig. 1:**
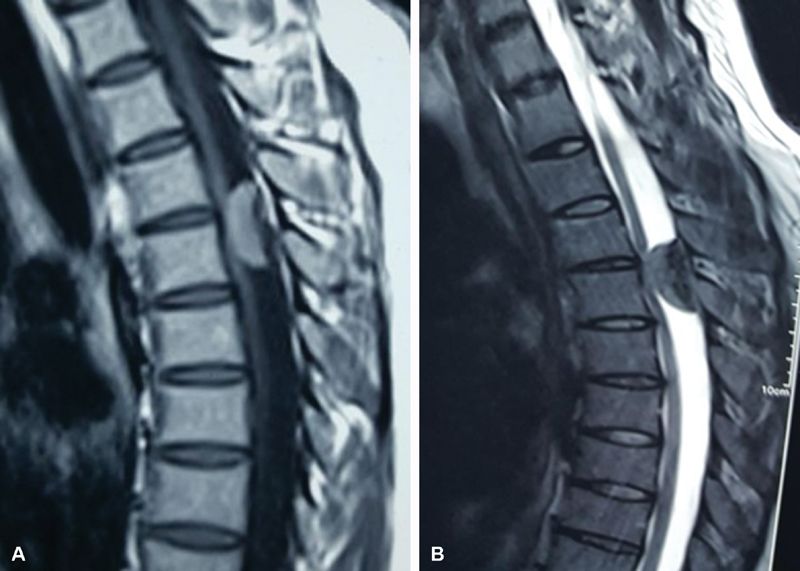
( **A** ) T1-weighted dorsal spine magnetic resonance imaging (MRI) showing spinal meningioma opposite T4–T5 with hypointense signals denoting calcifications. ( **B** ) T2-weighted dorsal spine MRI showing spinal meningioma opposite T4–T5 with hypointense signals denoting calcifications.

**Fig. 2:**
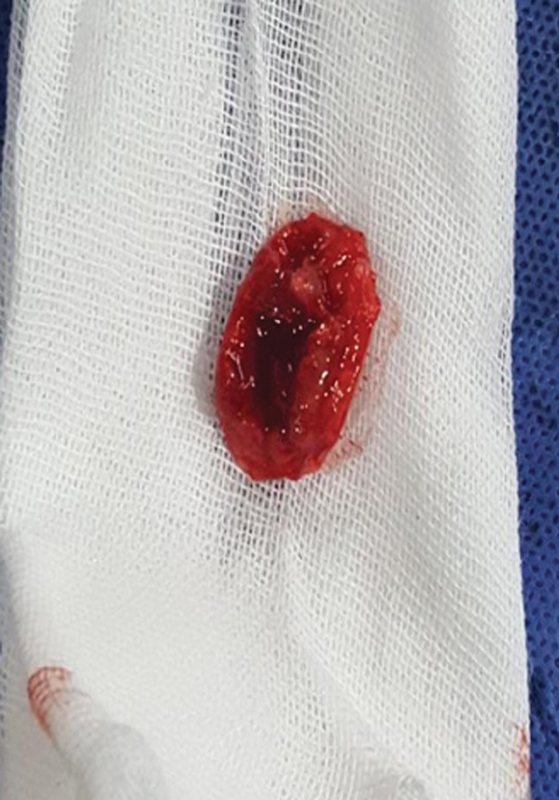
Intraoperative image of excised ossified meningioma.

**Fig. 3:**
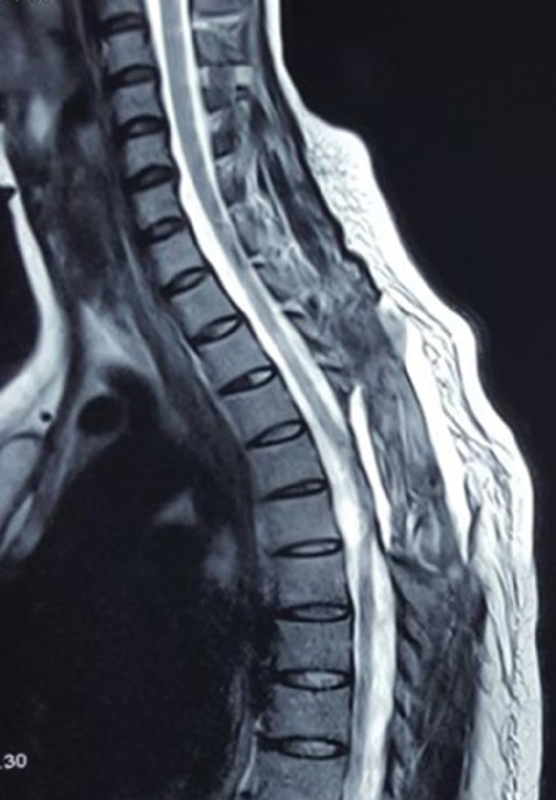
Postoperative T2-weighted MRI showing complete tumor removal. MRI, magnetic resonance imaging.

**Fig. 4:**
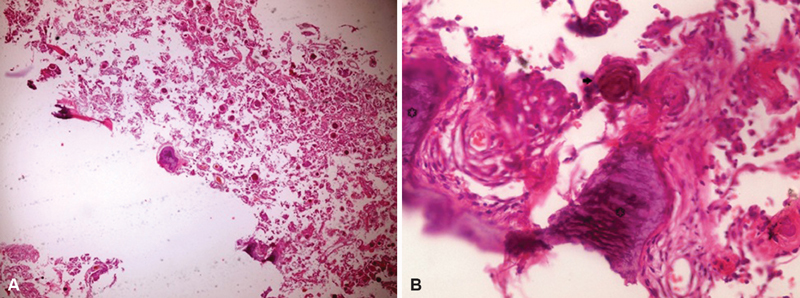
( **A** ) Photomicrograph showing meningothelial cells, calcified psammoma bodies, and scattered foci of ossification (hematoxylin and eosin [H&E], ×40). ( **B** ) Photomicrograph showing areas of irregular, calcified bone trabeculae (star) and calcified psammoma body (arrow; H&E, ×400).

A 22-year-old female presented with progressive heaviness in the lower limbs with gait disturbance, paresthesia of the lower limbs, and sphincter disturbances for approximately 5 weeks. Neurological examination revealed motor power grade of 3 for all muscle groups of the lower limbs and hypoesthesia with a sensory level below T4. Reflexes were exaggerated with positive Babinski and pathological reflexes. Radiological workup including magnetic resonance imaging (MRI) of the dorsal spine showed an intradural extramedullary spinal meningioma opposite the T4 to T5 level with hypointense signals on T1- and T2-weighted images of the tumor that indicated calcification ( Fig. 1A and B ). Under general anesthesia and intraoperative neuromonitoring, the patient was positioned in the prone position and a midline-skin incision was made, followed by two levels of total laminectomy without compromising the facet joints. Further, a linear midline incision was made through the relatively tough dura. Early identification of tumor poles and cerebrospinal fluid (CSF) drainage allowed fine neural retraction. Total tumor excision ( Figs. 2 and 3 ) was performed using standard microsurgical techniques. Dural attachment was coagulated, and appropriate hemostasis and watertight dural closure were performed. The patient's neurological status improved to full motor power after postoperative 6 weeks using a rehabilitative physiotherapy protocol. Histopathological examination revealed ossified meningioma ( Fig. 4A and B ).

## Discussion and Review of Literature

Including this report, a total of 33 cases of ossified spinal meningioma have been published till 2019 according to PubMed ( Table 1 ). For this condition, female predominance has been clearly noted (female, 31; male, 2). The mean age of patients with this condition is 42.6 years, with the youngest patient being 15 years old. The tumor was located in the thoracic spine in all patients except four (cervical region, three; lumbar region, one). Further, the tumor was located posterior to the spinal cord in 18 patients.

**Table 1:** Review of ossified meningioma cases

Year	Author	Number of cases	Age and sex	Location	Level
1928	Rogers 19	1	16/F a	Dorsal	T c 9
1972	Freidberg 20	1	69/F	Ventral	T1–2
1993	Niijima et al 21	2	75/F–75/F	Dorsal, ventral	T8–9, T9–10
1994	Kitagawa et al	2	60/F–45/F	Dorsal, lateral	T7–8, c2
1996	Nakayama et al	1	74/F	Dorsal	T9
1999	Huang et al 22	1	73/F	Lateral	T5
2001	Naderi et al 23	1	15/F	Dorsal	T4
2006	Liu et al 24	1	70/F	lateral	T11
2009	Hirabayashi et al 25	1	82/F	Dorsal	L3
2009	Uchida et al	1	76/F	Ventral	T12
2010	Licci et al 26	1	58/F	Lateral	T6
2013	Chotai et al 27	1	61/F	Dorsal	T4–T5
2013	Ju et al 28	1	61/F	Lateral	T9–10
2013	Taneoka et al 29	1	78/F	Dorsal	T9
2014	Yamane et al 30	1	61/F	Ventral	T12
2014	Chan et al 31	1	64/F	Dorsal	T9–10
2015	Alafaci et al	9	Mean age 59 years/8F–1m b	4 ventral, 4 dorsal, 1 lateral	7 thoracic, 2 cervical
2016	Demir et al 32	1	26/F	Dorsal	T9–t11
2016	Xia et al 33	1	90/M	Dorsal	T10–T11
2016	Kim et al 34	2	51/F– 77/F	Dorsal, lateral	T4, t9
2018	Prakash et al 35	1	60/F	Dorsal,	T7–T8

a Female.

b Male.

c Thoracic.

Spinal meningiomas arise from the arachnoid villi of spinal nerve roots and are located within the intradural space in most cases. 5 Except complete psammomatous transformation, the pathogenesis of ossification remains unclear; some theories suggest that meningioma ossification occurs secondary to metaplasia of arachnoid or interstitial cells on exposure to osteoblast-transforming factors such as Sox9. 6 The selection of the initial site of mineralization and mode of calcification in psammoma bodies is attributed to hydroxyapatite crystal precipitation within the bodies which result in the formation of large psammoma bodies. Then, collagen fibers surrounding the calcified bodies accumulate deposits of apatite crystals, forming larger psammoma bodies. 7 In addition, estrogen deficiency is hypothesized to enhance the process of calcification in areas containing necrotic fibroblasts and increased number of collagen fibrils. 8 9 Uchida et al reported that premature arachnoid cells with pluripotency differentiate into metaplastic cells and lead to bone formation. 8

The clinical features of ossified spinal meningiomas include motor, sensory, and sphincter dysfunctions, which exhibit different phenotypes according to the tumor location and neural compression. 10

Ruggeri et al observed a statistically significant relationship between postoperative neurological status and the degree of meningioma ossification wherein surgical morbidity increased with calcified and ossified tumors. 11 Detection of ossification is important during preoperative planning and preparation for safe tumor resection. Although MRI is considered as the best noninvasive neuroimaging technique, it cannot detect small amounts of calcification. High-signal areas on computed tomography (CT) are the most important radiological features for ossification detection. 12

The surgical strategy for ossified meningiomas differs from that for other classical cases of meningiomas because central tumor debulking can be challenging. Using an ultrasonic surgical aspirator is helpful for hard tissue removal, including bone removal; however, it may result in neurovascular damage. This method can be safely used at the tumor periphery. Meticulous microsurgical dissection between the pia mater and tumor surface can facilitate en bloc tumor removal. 13

When attempting a complete resection, wide resection of the dural attachment tends to reduce the rate of recurrence; however, dural coagulation can also be performed. 14 Splitting the dura mater into inner and outer layers is a simple technique that does not require complicated duraplasty with fascia or artificial dura. 15 The use of intraoperative neuromonitoring techniques to assess motor evoked potentials (MEPs) and somatosensory evoked potentials (SSEPs) helps in reducing postoperative iatrogenic neurologic deficits. 16

Approximately 90% of spinal meningiomas can be surgically resected with Simpson's grade-1 resection. The rate of recurrence during long-term follow-up reportedly ranges from 4 to 10%. 10

Adjunctive radiation therapy is considered for cases requiring subtotal resection; those with recurrent meningiomas, anterior tumor location, and en plaque and calcified meningiomas; and those wherein the surgical risk is extremely high, given patients' comorbidities and tumor location. 17 18

## Conclusion and Recommendations

Spinal meningiomas with a hypointense signal on MRI should indicate surgeons about the possibility of calcified or ossified meningiomas. The authors recommend the use of preoperative CT to detect sites of ossification. Intraoperative neuromonitoring of MEPs and SSEPs, use of a wide surgical corridor with total laminectomy, wide dural opening, identification of upper and lower poles, and early CSF drainage are helpful in decreasing neural retraction, thereby facilitating safe total resection. The authors recommend dural resection or coagulation to reduce the rate of recurrence and to avoid redo surgeries.
